# Detection method of PPE wearing and small target positioning for offshore operators: The improved YOLOv11 model and targets recognition

**DOI:** 10.1371/journal.pone.0335891

**Published:** 2025-11-17

**Authors:** Xiaosai Guo, Shihai Zhang, Chongnian Qu, Qingzheng Zhang

**Affiliations:** School of Mechanical Engineering, Tianjin University of Technology and Education, Tianjin, China; The University of British Columbia, AUSTRALIA

## Abstract

Focusing on the practical challenges of insufficient samples, incomplete categories, and low detection accuracy (particularly for small targets) in Personal Protective Equipment (PPE) wearing condition monitoring for operators in offshore environments, this research investigates PPE targets detection for offshore operators using an improved YOLOv11 model. The optimized model integrates the time-frequency features enhancement module (Spatial Pyramid Pooling-Fast, SFEAF) into the model’s backbone network, employs a statistical-driven dynamic gating attention module (Token Statistics Self-Attention, TSSA) to refine attention weight distribution in the original C2PSA module, and incorporates a Normalized Wasserstein Distance (NWD) loss function. These modifications collectively enhance the model’s capability to detect PPE targets for offshore operators. To mitigate missed detection problem of small targets such as earplugs and gloves, a cascaded network of YOLOv11 and YOLOv11-Pose models is proposed for small targets detection. The solution involves extracting human key points through YOLOv11-Pose model, constructing spatial constraint regions via two-point area positioning method, enhancing small target features through localized region cropping and normalization, and performing secondary detection on refined regions using YOLOv11 model. The ablation experiments show that the mAP@0.5 value of the optimization model has been improved by 1.8 percentage points compared to the original model for all targets, and the precision rates for both positive and negative samples of small targets—earplugs and gloves—are respectively improved by 5.2%, 4.2%, 0.2%, and 3.7%. The superiority of the optimization method has been proved. Furthermore, secondary detection experiments on small targets yielded an average Missed Detection Recovery Rate (MRR) of 56.64%, and the effectiveness of the multi-model cascaded detection method has been verified.

## Introduction

The operational environments of offshore platforms and vessels have the characteristic of confined spaces, densely arranged equipment, harsh conditions, and concentrated safety hazards. Correctly wearing Personal Protective Equipment (PPE) is the last protective line of personal safety for offshore operators. However, there are a few personnel demonstrating inadequate safety awareness at offshore worksites and occasionally access operational zones without complete PPE, which poses significant safety risks. The traditional manual supervision mode of PPE wearing condition has problems such as insufficient real-time and continuity, and limited coverage.

Currently, the PPE wearing condition detection based on vision and AI technologies has achieved successful deployment in construction sites and industrial workshops. Considering the unique characteristics of offshore working environments, the following problems need to be adjusted and optimized appropriately.

(1) In current applications, the PPE samples utilized to train the deep learning target recognition models mainly come from working environments such as onshore buildings and workshops. Due to the dataset differences, the detection accuracy of the trained model is low for the PPE targets of offshore operators.
(2) Comparing with the land working environment, the offshore operators require an expanded range of PPE, which include safety helmet, protective clothing, safety shoes, goggles, earplugs, earmuffs, and gloves, and each category exhibits multiple variants.
(3) Based on the complexity of the offshore operational environment structure, there are issues such as target occlusion and small targets in the monitored images or videos, and the targets of earplugs and gloves are very difficult to be detected accurately.

To address above issues, this study systematically investigates PPE targets detection method for offshore operators using the YOLOv11 model, while develops a target localization and secondary detection method based on the cascaded network of YOLOv11 and YOLOv11-Pose models for the difficult detection targets of earplugs and gloves.

## Related work

Recent advancements in machine vision and artificial intelligence have motivated researchers to conduct extensive studies on intelligent detection for PPE wearing condition. Existing studies on PPE detection can be broadly categorized into Transformer-based methods and YOLO-based methods.

Transformer-based methods: Xu et al. proposed Dynamic Multi-Scale Detection Transformer (DMS-DETR) for efficient PPE detection, which significantly improves accuracy for small and occluded objects [1]; Chen et al. proposed a novel Swin transformer-based feature extraction network incorporating a deformable attention mechanism, which enhanced multi-scale feature contextual awareness while preserving computational efficiency, thereby improving helmet detection accuracy [2]; Xiang et al. developed MAE-NAS using a MobBlock variant integrated with a multi-scale Swin Transformer module, achieving both real-time helmet detection and high accuracy [3]; Riaz et al. employed a Swin-UNet-based self-attention mechanism that combines global and local feature extraction for PPE detection, ensuring robust generalization and adaptability under diverse field conditions [4]; Chen et al. proposed ladder-type multi-attention network (LMNet), a hybrid model based on YOLOv5 and Residual Transformer 3D-spatial Attention Module (RT3DsAM), which utilizes high-resolution feature representation to improve the detection of small aerial targets [5].

YOLO-based methods: Wang et al. developed an efficient video-based multi-target tracking algorithm by integrating the DeepSort tracking algorithm with an improved YOLOv7 object detection algorithm, achieving efficient PPE detection and precise tracking [6]; Barlybayev et al. created a high-quality dataset to evaluate the detection accuracy of different YOLOv8 models on three PPE categories including helmets [7]; Ferdous et al. proposed an automated PPE detection system based on YOLOX and evaluated it on a custom dataset (four colored hardhats, vest, safety glass, CHVG) containing four PPE categories [8]; The YOLO-EfficientNet hybrid architecture proposed by Lee et al. enhanced helmet recognition accuracy through a two-stage detection-classification model [9]; Iannizzotto et al. proposed a framework combining DNN-based object detection with manual verification for identifying incorrect PPE usage, obtaining high detection accuracy with limited datasets [10].

Although existing studies have improved PPE detection in specific contexts, most focus on construction or indoor industrial environments and typically target single types of equipment rather than comprehensive PPE suites. Moreover, small objects such as earplugs and gloves remain challenging to detect, particularly in offshore environments characterized by occlusion and complex backgrounds. To address these limitations, this study proposes an improved YOLOv11-based model with optimizations in the backbone network, attention mechanism, and loss function. The detection scope has been expanded to include five PPE categories (helmets, earplugs, protective clothing, gloves, and safety shoes). Furthermore, a multi-model cascade framework combining the improved YOLOv11 with YOLOv11-Pose is introduced to enhance the detection accuracy of small PPE items (earplugs and gloves), addressing the practical requirements of PPE monitoring in offshore platform settings.

## The optimization for YOLOv11 model

YOLOv11 is an efficient object detection algorithm developed by the Ultralytics team [11]. It incorporates the Cross Stage Partial with kernel size 2 (C3K2) and the Cross Stage Partial with Spatial Attention (C2PSA). The C3K2 [12]module optimizes information flow within the network by splitting feature maps and applying a series of small-kernel convolutions (3 × 3), thereby reducing computational overhead while preserving the model’s capacity to capture essential image features. Furthermore, the YOLOv11 backbone integrates a C2PSA module after the Spatial Pyramid Pooling - Fast (SPPF) layer [13]. This module employs two Partial Spatial Attention (PSA) mechanisms to enhance focus on semantically salient regions of the image. By integrating these two core components to improve both detection accuracy and computational efficiency, the YOLOv11 model has demonstrated superior performance across various complex detection tasks [14].

### The backbone network optimization based on SFEAF module

**Fig 1:**
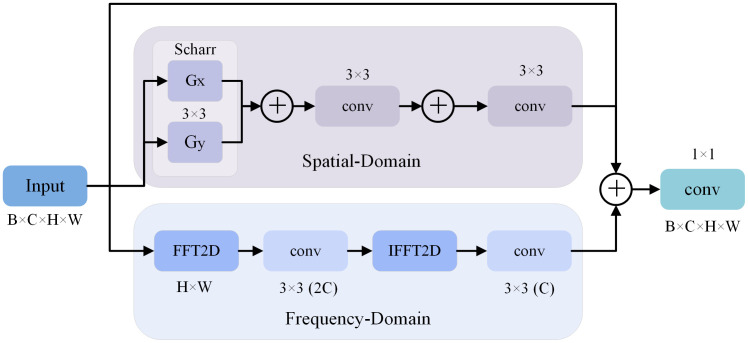
The SFEAF module structure.

The original YOLOv11 model primarily relies on CNN to extract local features in the time domain. The CNN with local receptive field characteristic tends to capture low-frequency semantic information (e.g., target contours), but its sensitivity to high-frequency details (e.g., edges and textures) is insufficient, which results in incomplete feature representation of small targets or complex textured targets. On the contrary, the high-frequency information can be enhanced through filtering in the frequency-domain feature extraction method, and the global energy distribution of images can be reflected [15]. To improve the detection accuracy of YOLOv11 model for PPE targets, we propose the Spatial-Frequency Edge-Aware Fusion (SFEAF) dual-path heterogeneous architecture by integrating time and frequency domains. The structure of SFEAF module is shown in Fig 1.

The time domain branch in Fig 1 focuses on capturing pixel-level spatial features related to local and regional characteristics. To enhance the edge perception capability of spatial domain paths and reduce computational errors in gradient magnitude caused by asymmetric Sobel kernel coefficients in rotational scenarios, the Scharr operator replaces the traditional Sobel convolution for gradient magnitude calculation, employing 3 × 3 convolutional kernels in both horizontal (Gx) and vertical (Gy) directions to extract edge features. This results in gradient computations that more closely approximate theoretical differential values. Additionally, the two-stage convolutional layers are used to perform nonlinear mapping for edge features in the time branch, and the skip connections is introduced to alleviate gradient vanishing.

The frequency domain branch in Fig 1 emphasizes global frequency characteristics of entire images. Firstly, input feature maps are transformed into the frequency domain by using Fast Fourier Transform (FFT) [16], the FFT is applied without additional sub-windows. Secondly, the frequency-domain features are filtered through convolutional layers whose weights are learned via backpropagation to enhance high-frequency components (e.g., edges) and suppress low-frequency noise. The filtered features are then decomposed into real and imaginary parts. Thirdly, the filtered features are reconstructed into the time domain by inverse FFT, and the channel dimensions of time domain and frequency domain are aligned by convolutional layers.

In totally, the time domain channel is used to enhance local edge features extraction through Scharr convolution, while the frequency domain channel is used to capture global spectral features using FFT. The time-frequency dual channel is introduced into the YOLOv11 model backbone network to address the issue of “local-global” feature imbalance in PPE targets detection and enhance the feature representation capability of the PPE targets.

### Integration of the TSSA attention mechanism

**Fig 2:**
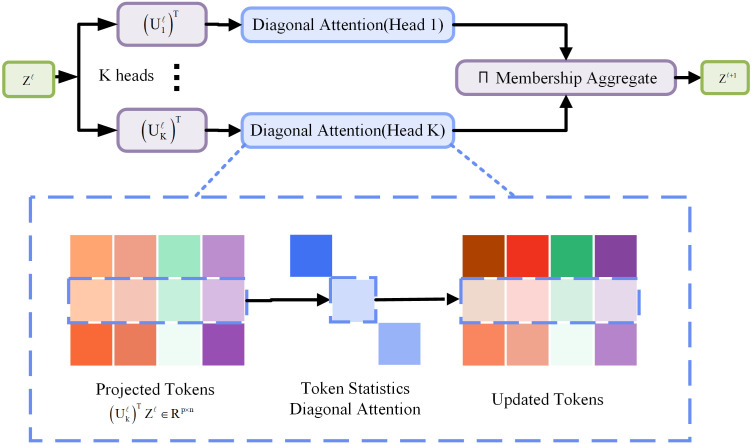
Token statistics self-attention.

The attention mechanism in the C2PSA module of the original YOLOv11 model relies on pairwise similarity calculation, and the problem of quadratic complexity is inevitable. In the contrary, Token Statistics Self-Attention (TSSA), an attention mechanism using maximum coding rate reduction (MCR²) [17], employs a linear-complexity design based solely on global statistical measures. As illustrated in Fig 2, the original attention mechanism in the C2PSA module is replaced by TSSA module to enhance the overall performance and deployment efficiency of YOLOv11 model, and the missed and false detection for small targets can be reduced predictably. The TSSA dynamically generates gating weights by using global statistical measures (e.g., second-order moments) method, the required pairwise similarity computation of traditional attention mechanisms can be avoided, and the computational complexity can be reduced from O(n²) to O(n). Furthermore, TSSA demonstrates enhanced robustness against complex backgrounds and occluded targets through a variance-driven feature suppression mechanism.

Given the input feature matrix *Z*=[*z*_1_,...,*z*_*n*_] ∈ℝ^*d*×*n*^, *Z*_*j*_ represents the *j-*th token. Its first-order moment *μ* and second-order moment *σ*^2^ can be defined as:


$$\mu =\frac{\text{1}}{n}\sum_{i=1}^{n}z_{j},\begin{array}{cc} {}*20c & \sigma^{2} \end{array}=\frac{\text{1}}{n}\sum_{i=1}^{n}\left(z_{j}-\mu \right)\circ (z_{j}-\mu )$$ (1)

where i denotes element-wise multiplication.

Based on these global statistics, TSSA starts from the compression term of MCR^2^, introduces a variational upper bound, and constructs the attention operator. Its functional definition is:


$$R_{c,f}^{var}(Z,\Pi |\{U_{k}\})=\frac{\text{1}}{\text{2}}\sum_{k=1}^{K}\frac{n_{k}}{n}\sum_{i=1}^{p}f(\frac{\text{1}}{n_{k}}(U_{k}^{T}Z\;diag(\pi_{k})\;Z^{T}U_{k}{)}_{ii})$$ (2)

Where Π ∈ ℝ^*n×K*^ represents the assignment matrix, π_*k*_ is its *k*-th column, *n*_*k *_= ‹*π*_*k*_,1›;*U*_*k *_∈ ℝ^*d×p*^ represents the *k*-th subspace basis, diag(.) converts a vector to a diagonal matrix, and f(.) is a convex spectral function (such as *f*(*x*)=log(1+(*d*/*ε*^2^)*x*)), with ∇*f* indicating its derivative.

Taking the gradient of this variational objective with *Z* respect to the parameters.


$$\nabla_{Z}R_{c,f}^{var}(Z,\Pi |\{U_{k}\})=\frac{\text{1}}{n}\sum_{k=1}^{K}U_{k}\;diag(\nabla f\left[\frac{((U_{k}^{T}Z{)}^{\circ }(U_{k}^{T}Z))\cdot \pi_{k}}{n_{k}}\right])\;U_{k}^{T}Z\;diag(\pi_{k})$$ (3)

This Equation (3) indicates that the sensitivity of the objective function with respect to each token representation is primarily influenced by the second-order statistics and their projection onto the subspace basis. Additionally, by applying a gradient descent step with step size *τ* to this gradient, the update operator for the TSSA layer is derived as:


$$Z^{+}=Z-\frac{\tau }{n}\sum_{k=1}^{K}U_{k}\;D_{k}(Z|U_{k})\;U_{k}^{T}Z\;diag(\pi_{k}),\;D_{k}(Z|U_{k}):=diag(\nabla f\left[\frac{((U_{k}^{T}Z{)}^{\circ }(U_{k}^{T}Z))\cdot \pi_{k}}{n_{k}}\right])$$ (4)

where *D*_*k*_ (*Z* | *U*_*k*_) denotes the diagonal weight matrix generated from the current second-order statistics. Therefore, it can be concluded that TSSA effectively represents an unrolled (white-box) optimization step for the variational compression objective. With an appropriately chosen step size*τ*, the method can monotonically decrease the value of the objective function.

Since TSSA leverages second-order statistics to compute dynamic gating weights along the channel dimension, replacing the traditional pairwise similarity calculation in self-attention, it is employed to enhance the overall performance and deployment efficiency of the YOLOv11 model. This modification aims to mitigate the issues of missed and false detections, particularly for small target recognition. Specifically, TSSA replaces the attention mechanism in the C2PSA module and is integrated at the end of the YOLOv11 Backbone (post-SPPF). The key hyperparameters for the TSSA attention mechanism are as follows: the core output channel number is set to 1024, the feature dimension is 1024, the expansion rate e is 0.5, and 8 attention heads, consistent with the original configuration, are used. The QKV bias is set to False, and no additional Dropout or bias terms are applied.

### Introduction of the NWD loss function

The original CIOU loss function in YOLOv11 exhibits excessive sensitivity to positional deviations of small targets. Minor positional deviations can induce instability in positive sample assignment and discontinuity in bounding box regression gradients during training, and the model detection performance degrades significantly when the average dimensions of targets less than 16 × 16 pixels. To address this limitation, the Normalized Wasserstein Distance (NWD) [18] is introduced as the loss function to establish the NWDet module. This approach alleviates the inherent limitations of CIOU by modeling bounding box distribution characteristics, thereby enhancing model robustness for small targets [19].

The core innovation of NWD lies in representing bounding box $R=\left(c_{x},c_{y},w,h\right)$ as 2D Gaussian distributions N(μ,Σ) and utilizing distribution similarity metrics instead of traditional geometric overlap measures, where $\left(c_{x},c_{y}\right)$ denotes the central coordinates, w and h respectively represent width and height of bounding box. The mean vector corresponding to the central coordinates is given by Equation (5).


$$\mu ={\left[{\text{c}}_{\text{x}},{\text{c}}_{\text{y}}\right]}^{T}$$ (5)

The w and h are transformed into a covariance matrix through an inscribed ellipse formulation, and the pixel-wise distributions within the bounding box characterized as formulated in Equation (6).


$$\Sigma =diag\left({\left(w/2\right)}^{2},{\left(h/2\right)}^{2}\right)\mathrm{\backslash ]}$$ (6)

To quantify the displacement magnitude between the center points of predicted and truth bounding boxes, the spatial coverage difference of predicted and truth bounding boxes is calculated by establishing two Gaussian distributions $N_{a}$ and $N_{b}$, whose similarity is measured via the second-order Wasserstein distance as defined in Equation (7):


$${W_{\text{2}}}^{\text{2}}(N_{a},N_{b})=∥\mu_{a}-\mu_{b}{∥}_{2}^{2}\;∥\Sigma_{a}^{1/2}-\Sigma_{b}^{1/2}{∥}_{F}^{2}$$ (7)

In the right side of Equation (7), the first term represents the Euclidean distance between mean vectors and it quantifies the central displacement magnitude of bounding boxes, the second term corresponds to the Frobenius norm of the covariance matrix square roots and it quantifies the difference of scale and geometry. To construct a differentiable similarity metric, the Wasserstein distance is further mapped to the 0,1 interval through exponential normalization, which is formulated in Equation (8):


$$NWD(N_{a},N_{b})=exp\left(-\frac{{W_{\text{2}}}^{\text{2}}(N_{a},N_{b})}{\alpha \times \sqrt{E[w^{\text{2}}+h^{\text{2}}]}})\right)$$ (8)

In Equation (8), the term $\alpha \cdot \sqrt{E[w^{2}+h^{2}]}\backslash )$ represents the dataset-adaptive scaling factor to eliminate scale dependence. By Equation (8), the optimization objects are transformed into “minimizing discrepancy” from “maximizing similarity” thereby aligning with gradient descent frameworks. Consequently, the NWD loss function is defined as Equation (9).


$${\text{L}}_{\text{NWD}}=\text{1}-\text{NWD}$$ (9)

**Fig 3:**
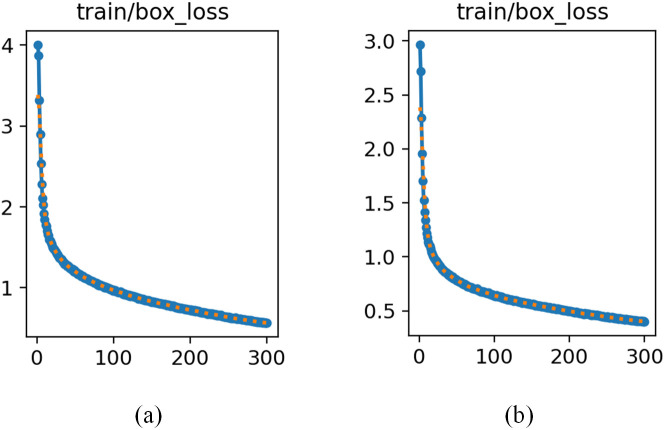
Experimental comparison of loss curves. (a)CIOU (b)NWD.

Comparing the training results of the NWD loss function with the original loss function CIOU, Fig 3 is obtained:

Due to the smooth gradient characteristics of NWD loss function, the vanishing gradient problem inherent in IoU under non-overlapping or fully contained condition is effectively mitigated. Furthermore, the discriminability for ambiguous boundaries of small targets can be enhanced through distribution matching mechanisms.

### Enhanced YOLOv11 architecture

**Fig 4:**
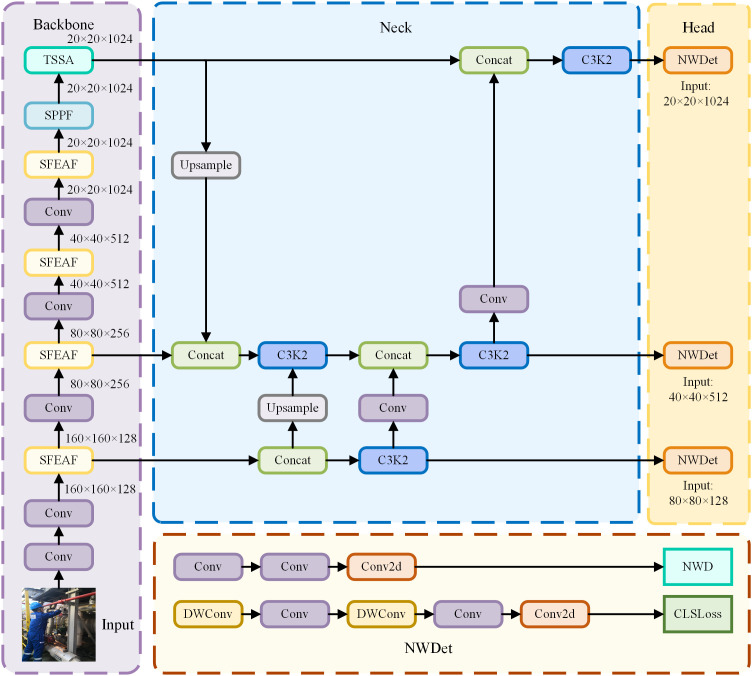
The improved YOLOv11 model structure.

The YOLOv11 architecture consists of three primary components: Backbone, Neck, and Head. The Backbone is responsible for extracting multi-level feature maps from the input image, while the Neck samples and fuses feature maps from different levels, enabling the model to effectively capture multi-scale information and transmit it to the Head for prediction. The Head processes the feature map received from the Neck, fusing high-level semantics and low-level image features through upsampling and downsampling, thereby enhancing the model’s multi-scale object detection capabilities. Ultimately, the network outputs the bounding box, confidence score, and class probability for the detected targets. Integrating above optimization methods, the improved YOLOv11 module is shown in Fig 4.

### Small target detection via YOLOv11/YOLOv11-pose cascade

Based on the improved YOLOv11 model in Fig 4, the detection accuracy for various PPE targets on offshore operators can be improved in theory. All the same, our testing indicates that the the detection accuracy for small targets (e.g., earplugs and gloves) are difficult to meet the application requirement. To address this limitation, we develop a cascaded detection framework integrating the improved YOLOv11 and the YOLOv11-Pose models for difficult detection small target.

### Multi-model cascade framework

**Fig 5:**
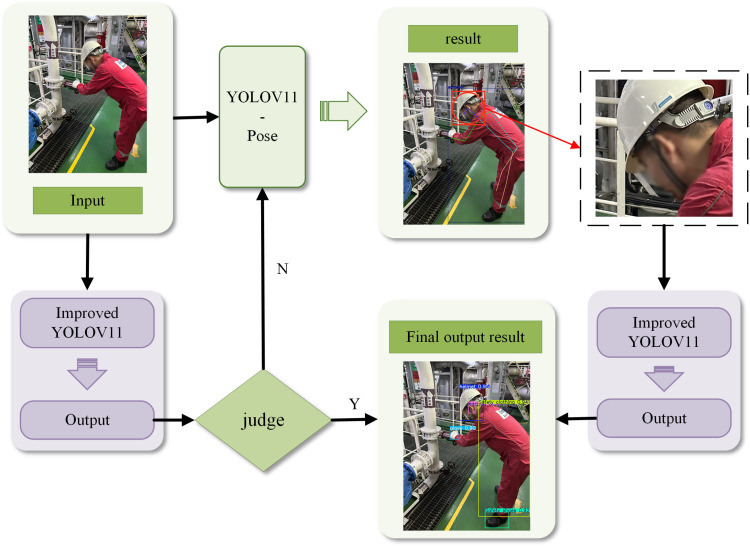
Multi-model cascaded detection framework for small PPE targets.

It is shown that the human pose recognition model can detect the position information of human key points in a given image or video, and there is also a strong correspondence among the PPE and key points of the human body. For example, the earplugs correlates with auricular key points, the gloves correlates with wrist joints, and so on. Leveraging these correlations, this study proposes a multi-model cascaded detection framework as illustrated in Fig 5.

In Fig 5, the input image is detected firstly by the improved YOLOv11 model, if one or more person targets and their complete corresponding PPE targets are detected, the normal detection results will be outputted.

In contrary, if one or more PPE targets corresponding to a person are not detected in the picture, the YOLOv11-Pose model will be activated to detect the key points of the human body. For the key points corresponding to the undetected PPE targets, the two-point region positioning method is used to extract the local image region, and then the YOLOv11 model is reapplied to detect the trimmed local area map again. If the corresponding PPE target is detected, the normal detection result will be generated, otherwise, an alarm message will be output.

Based on the above analysis, the improved YOLOv11 serves as the detection backbone and is integrated as a component within a multi-model cascade framework. This framework, in turn, coordinates the collaboration among multiple models—including the improved YOLOv11 and YOLOv11-Pose—to facilitate object detection in complex scenarios.

### Human posture network

Among mainstream human pose estimation algorithms, YOLOv11-Pose, as an extension of the single-stage detection framework, integrates the C3k2 module and spatial attention mechanism, and constructs an end-to-end joint optimization framework for feature extraction and pose estimation. Its improved parallel design not only improves the spatial attention efficiency for local human parts but also achieves a balance between computational efficiency and accuracy through a lightweight parameter structure. Meanwhile, YOLOv11-Pose combines the context-adaptive characteristics of YOLOv11 with improved skeleton topology modeling ability, and the problems of dense occlusion and posture variation in industrial scenes are effectively solved, which fully meet the dual requirements of robustness and engineering deployment in offshore operation scenarios.

**Fig 6:**
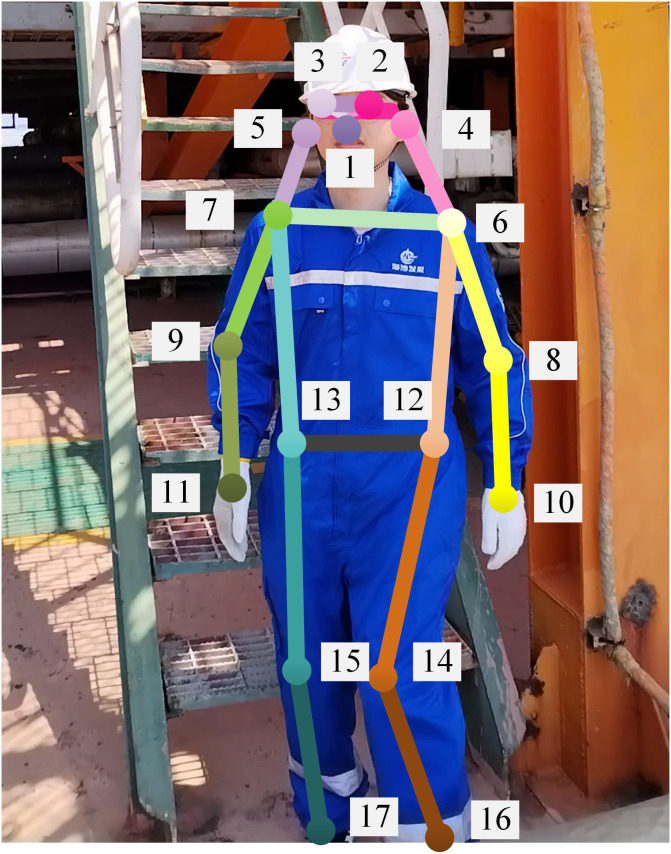
Distribution of key points of human body.

The relationship between PPE targets and the 17 bone key points commonly used in human posture recognition is shown in Fig 6 and Table 1.

**Table 1:** Association between key points and PPE.

PPE	Protective area	Key points
**Safety Helmet**	Head	Eyes, Ears, Nose
**Protective Suit**	Body	Shoulders, Elbows, Waist, Knees
**Earplugs**	Ears	Ears
**Gloves**	Hands	Wrists
**Safety Shoes**	Feet	Ankles

The key points detected by the YOLOv11-Pose model can be used as spatial anchor points for local positioning detection, and guiding the attention mechanism toward a predetermined area. Different key points can be individually or jointly located a certain detection area.

### Two-point region localization method

In the paper, based on the key points information output by the human pose estimation model, two specific attention regions are designed: the ear region (K) and the hand region (R), here, k_*i*_ ∈ K represents the *i*-th ear detection region, and r_*i*_ ∈ R represents the *i*-th hand detection region. The region generation algorithm adopts the two-point positioning method, and its mathematical definition is as follows.

Let p_base_ represent the neighboring key point of the target to be detected and p_ref_ represent another nearby reference key point, and a circular detection area is constructed with p_base_ as the center, as shown in Equation (10).


$$C(p_{base},r)=\{(x,y)|(x\text{-}x_{base}{)}^{\text{2}}+(y\text{-}y_{base}{)}^{\text{2}}\leq r^{\text{2}}\}$$ (10)

Taking the ear region as an example, the coordinates of the left ear are defined as p_le_ (x_*le*_, y_*le*_), and the coordinates of the left shoulder are p_ls_ (x_*ls*_, y_*ls*_). similarly, the coordinates of the right ear and right shoulder are p_re_ (x_*re*_, y_*re*_) and p_rs_ (x_*rs*_, y_*rs*_) respectively. Taking the left ear and left shoulder as an example, the distance between them can be expressed as in Equation (11).


S=[xmin,ymin,xmax,ymax] (11)

**Fig 7:**
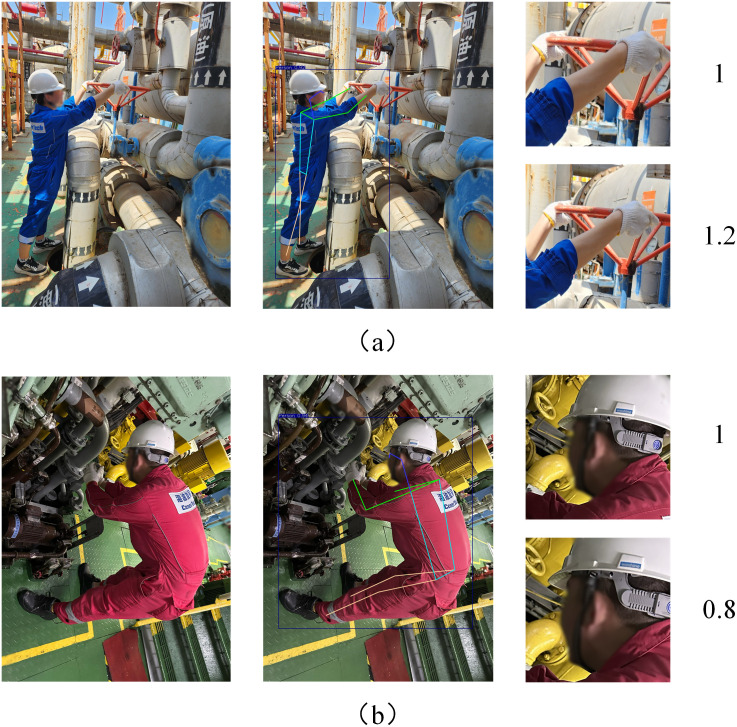
Comparative schematic of dynamic radius scaling factor values. (a) *β* = 1.2 (b) *β* = 0.8.

Considering the size variations of different PPE targets, a dynamic radius scaling factor *β* is introduced. For the ear region, due to the size of human ear is small, *β* is set to 0.8, which reduces background interference by reducing the area while ensuring that the earplug target is located within the attention zone. For the hand region, considering that localization errors of hand and elbow keypoints are inevitable in practical detection—potentially causing the target to not be fully contained within the region of interest—we set *β* = 1.2 to balance the coverage area and ensure target integrity within the attention zone. Through experimental validation, representative examples were selected to illustrate the cropping effects under different values of *β*, as shown in Fig 7.

The local circular radius quation considering the scaling factor can be expressed as $L=\beta \cdot L_{l}$.Taking the coordinates of p_base_ as the center and L as the radius, the local circular detection area can be gained. The circumscribing square of the circular detection area can be taken as the second detection area and the pixel coordinates of this square are expressed in Equation (12).


S=[xmin,ymin,xmax,ymax] (12)

$$\left\{ \begin{array}{c} x{\_}_{\min}=\max\left(0,x_{ear}-L\right)\\ x{\_}_{\max}=\min\left(W,x_{ear}+L\right)\\ y{\_}_{\min}=\max\left(0,y_{ear}-L\right)\\ y{\_}_{\max}=\min\left(H,y_{ear}+L\right) \end{array}\right.$$ (13)

**Fig 8:**
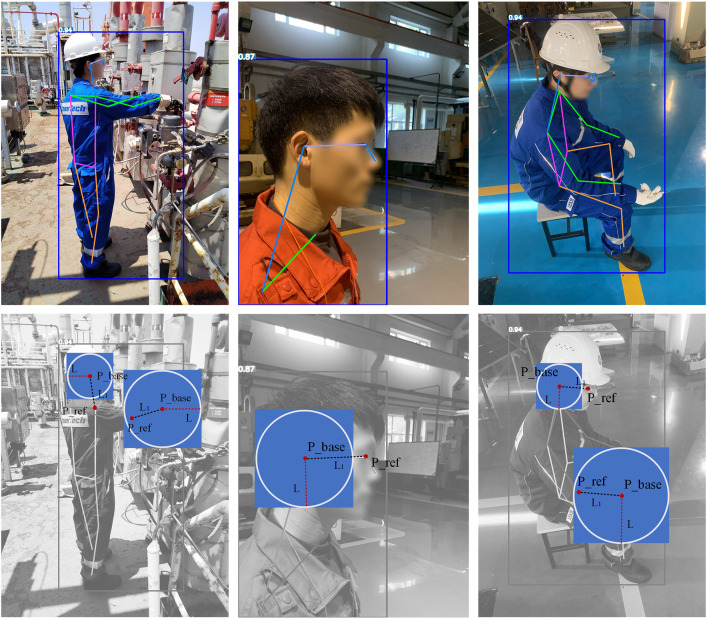
The schematic diagram of two-point positioning method. (a) Image example 1 (b) Image example 2 (c) Image example 3.

In Equation (13), W and H respectively represent the pixel width and height of the image to be detected. When the pixel coordinates of local square exceed the image boundaries, the image boundaries parameters are taken as the pixel coordinates local square. The principle of the two-point positioning method is illustrated in Fig 8.

Considering the potential occlusion of key points in real production environments, other adjacent key points of the detecting key point will be selected for regional radius calculation. For example, if the shoulder key point is occluded, the adjacent eye key point of the detecting ear key point will be selected. In addition, considering the condition of multiple human poses in a single image, the sets {K_i_} and {R_i_} are independently constructed for each detected individual based on individual key points information from the human pose estimation model, which ensures spatial independence in PPE targets detection among different offshore operators. Each attention region is tightly associated with a specific key point (ear/hand) so as to avoid erroneous cross-individual associations. The upper-left vertex coordinates (x_min_, y_min_) of each detecting region are extracted, and regions are numbered in ascending order of the x-coordinate to generate an ordered region set.

## Experimental results and analysis

### Experimental conditions and parameter setting

The experimental environment for training the model in this study uses Ubuntu 20.04.6 LTS as the operating system, with 32.0 GB RAM. The CPU is a 13th Gen Intel(R) Core(TM) i7-13700KF, and the GPU is an NVIDIA GeForce RTX 3090. The algorithm framework is PyTorch 2.2.2, the programming language is Python 3.10, and the GPU acceleration libraries are CUDA 12.1 and cuDNN 8.9.2.

The model training parameters are set as follows, the number of epochs is set to 300, the batch size is set to 8, the SGD optimizer is used, the initial learning rate is 0.01, the momentum of the learning rate is 0.937, the weight decay coefficient is set to 0.0005, and the input image size for the model is 640 × 640.

### Dataset construction and evaluation metrics

Based on the PPE commonly used by offshore operators, a basic image dataset of PPE targets for offshore operators (including positive samples—PPE are correctly worn, and negative samples—PPE are not or incompletely worn) was constructed through simulation shooting under the onshore and offshore operational environments. The basic dataset was collected using multiple smartphone models (Samsung Galaxy S22 Ultra and vivo X Fold3), with resolutions ranging from 4000 × 3000–3072 × 4096 pixels, and 4,423 site images were collected.

Furthermore, for the training set, data augmentation techniques—including image rotation, horizontal flipping, contrast adjustment, brightness variation, occlusion, and cropping—were applied stochastically, both individually and in composite manner. Comprehensive the image acquisition and augmentation methods, the final sample distribution is as follows: safety helmets (5824 samples, 17.16%), earplugs (4023 samples, 12.26%), protective clothing (6644 samples, 20.24%), gloves (8361 samples, 25.48%), and protective shoes (7963 samples, 24.46%). Small targets (earplugs and gloves) account for 37.74% of the dataset. The dataset was randomly divided into training, validation, and testing sets in a 7:2:1 ratio. Manual annotation was adopted for the training and validation data. The labels include: “helmet”, “n-helmet”, “safety clothing”, “n-safety clothing”, “earplug”, “n-earplug”, “glove”, “n-glove”, “safety shoes”, and “n-safety shoes”.

In order to evaluate the model detection effect, the frequently-used evaluation metrics of Precision (P), Recall (R), F1-score, Average Precision (AP) and mAP@0.5 are introduce in the paper. Wherein, P represents the proportion of actual positives among the positive predictions, R represents the proportion of actual positives correctly identified by the model, F1-score represents the harmonic mean of precision and recall, AP represents the area under the P-R curve, mAP@0.5 represents the mean AP of all samples in a category when the Intersection over Union (IoU) threshold is set to 0.5.

### Ablation experiment of PPE targets recognition

According to the optimization methods mentioned earlier, five groups of control experiments are designed and the ablation results are shown in Table 2. In Table 2, M0 refers to the original YOLOv11 model, M1 refers to the model in which the SFEAF architecture is introduced based on M0, M2 includes the TSSA-C2PSA module, and M3 incorporates the NWD loss function.

**Table 2:** The ablation experiment results.

Model	P	R	F1	mAP@0.5
**M0**	0.918	0.792	0.855	0.848
**M0 + M1**	0.921	0.795	0.858	0.858
**M0 + M2**	0.926	0.798	0.862	0.863
**M0 + M3**	0.924	0.813	0.869	0.861
**M0 + M1 + M2 + M3**	0.935	0.815	0.875	0.866

The evaluation metrics in Table 2 represent the average testing performance across all categories. As shown in Table 2, comparing with the original YOLOv11 model, all the detection accuracy for PPE targets has been improved in different degrees corresponding to three optimization methods, and the fusion of the three optimization methods has achieved the best detection accuracy.

In order to further verify the optimization methods for small targets detection, the detection performance on positive and negative samples of earplugs and gloves are listed separately in Table 3, where Y and N represent respectively positive and negative samples.

**Table 3:** The small target detecting results.

Modle	Earplug/ mAP@0.5		Glove/ mAP@0.5	
	Y	N	Y	N
**M0**	0.669	0.676	0.884	0.646
**M0 + M1**	0.674	0.640	0.873	0.641
**M0 + M2**	0.701	0.706	0.877	0.686
**M0 + M3**	0.718	0.700	0.871	0.637
**M0 + M1 + M2 + M3**	0.721	0.718	0.886	0.683

**Fig 9:**
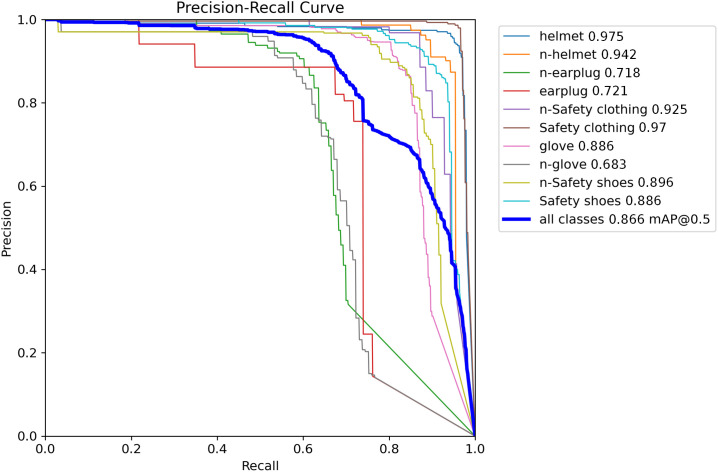
PR curve of the improved YOLOv11.

As can be seen from Table 3, comparing with the original YOLOv11 model, the detection accuracy of positive samples for earplug targets has been improved by introducing the SFEAF module into the backbone network. By introducing the TSSA-C2PSA module, the detection accuracy of three other types of targets has been improved, except for the glove positive sample. By introducing the NWD loss function, the detection accuracy of the model for positive and negative samples of earplugs can be significantly improved. It is indicated that the three optimization methods are highly complementary, and the detection accuracy for small PPE targets can be comprehensively improved by integrating three optimization methods. The PR curve is shown in the Fig 9:

However, the improvements yield relatively limited benefits for glove detection. This can be attributed to the considerable variation in color, morphology, and wearing posture of gloves, combined with the fact that both the SFEAF module and the TSSA attention mechanism are primarily designed to enhance feature representation for small targets or those with complex textures. In contrast to earplugs, which are more typical small targets, glove positive samples are generally larger in size and exhibit smoother textures. As a result, the original YOLOv11 model already achieves satisfactory detection performance for gloves, diminishing the marginal gains from the proposed enhancements.

### Efficiency comparison of the TSSA attention mechanism

To quantitatively evaluate the efficiency gains resulting from replacing the standard self-attention module with TSSA, the following metrics were adopted: Params (the total number of trainable parameters—weights and biases—in millions (M)), FLOPs (the number of floating-point operations in gigaunits (G) required for one forward pass/inference), Size (the storage size of the trained model weights in megabytes (MB)), and FPS (frames per second, indicating the number of images the model can process per second on specific hardware). The comparative experimental results are presented in Table 4.

**Table 4:** Efficiency comparative study.

Model	Params	FLOPs	FPS	size	mAP@0.5
**YOLOv11**	20.04	67.7	207	38.7	0.848
**YOLOv11 + TSSA**	19.71	67.5	215.7	38.0	0.863
**Improvement**	−0.33	−0.2	+15	−0.7	−0.015

As shown in Table 4, the TSSA attention mechanism not only improves accuracy but also reduces the overall parameter count of the model. Specifically, it achieves an increase of 15 FPS in detection speed, a reduction of 0.7 MB in model size, which demonstrates lower computational complexity and resource requirements. These improvements significantly enhance both the comprehensive performance and deployment efficiency of the YOLOv11 model.

### Cross-validation for improved YOLOv11 model

To provide a more robust evaluation of the improved YOLOv11 model’s performance and to validate the consistency of the experimental results, 5-fold cross-validation was employed. The dataset was randomly partitioned into five subsets, each containing approximately one-five of the total samples. The training and validation were performed over five rounds, where in each round, a different subset served as the validation set, and the remaining four subsets were used for training.

For each round, the model’s performance metrics (such as precision, recall, F1 score) were calculated, and the final evaluation metrics were derived by computing the mean and standard error across the five rounds to provide more stable performance estimates.

To assess the reliability and statistical significance of the experimental results, 95% confidence intervals (CIs) were calculated for each performance metric. The confidence intervals provide an estimate of the range within which the true value of the metric is likely to fall, based on the variation observed across the different folds of the cross-validation. The calculation of confidence intervals helps to quantify the uncertainty inherent in the performance estimates and provides a more robust interpretation of the results.

The 95% confidence interval for each metric was determined using the following formula:


$$CI=\overset{―}{X}\pm 1.96\times SE$$ (14)

Where $\bar{X}$ is the mean of the evaluation metric across the five folds, and SE is the standard error, which was calculated as:


$$SE=\frac{\sigma }{\sqrt{n}}$$ (15)

Here, *σ* represents the standard deviation of the metric values across the folds, and *n* is the number of folds (in this case, *n* = 5).

The experimental results of the improved YOLOv11 model under 5-fold cross-validation, together with the corresponding 95% confidence intervals are presented in Table 5.

**Table 5:** Improved YOLOv11 5-fold CV summary with 95% CIs.

Improved YOLOv11 Model	1	2	3	4	5	$\bar{X}$	95%CIs
**P**	0.937	0.92	0.908	0.91	0.934	0.922	[0.905, 0.938]
**R**	0.842	0.849	0.797	0.814	0.84	0.824	[0.801, 0.856]
**mAP@0.5**	0.889	0.886	0.872	0.878	0.883	0.882	[0.873, 0.890]

Table 5 shows that the model achieved a mean Precision of 0.922 (95% CIs [0.905, 0.938]), Recall of 0.828 (95% CIs [0.801, 0.856]), and mAP@0.5 of 0.882 (95% CIs [0.873, 0.890]). These values demonstrate that the performance of the improved model is consistently high across different data splits.

### Comparative experiment of different models

To further verify the superiority of the optimized model, several typical models are selected for comparison experiments. The datasets and experimental conditions are kept consistent across all comparison models. The results of the comparative experiments are shown in Table 6.

**Table 6:** The testing results of different model.

Model	P	R	F1	mAP@0.5
**SSD**	0.876	0.781	0.818	0.834
**Swin Transformer**	0.910	0.785	0.842	0.845
**YOLOv5m**	0.906	0.789	0.843	0.842
**YOLOv8m**	0.905	0.791	0.844	0.846
**YOLOv9m**	0.909	0.787	0.844	0.844
**YOLOv10m**	0.886	0.782	0.831	0.838
**YOLOv11m**	0.918	0.792	0.850	0.848
**Improved model**	0.935	0.815	0.871	0.866

As can be seen from Table 6, comparing with other earlier models, the selected YOLOv11m model performs best across all evaluation metrics for PPE targets detection. The applied optimization method further improved the detection accuracy of the YOLOv11m model. It is demonstrated that the selected basic model and optimization method are effective.

### Cascaded network experiments

#### The detecting effect evaluation metrics.

In order to evaluate the effectiveness of the cascaded detection method, the miss recovery rate (MRR) index is introduced and shown in Equation (16).


$$MRR=\frac{TR_{recovery}}{FN_{initial}}$$ (16)

In Equation (16), TP_*recovery*_ represents the number of missed detection targets that have been successfully corrected through secondary detection after locating the cropped area through key points. FN_*initial*_ represents the total number of missed detection targets of the improved YOLOv11 model, and the calculating method of FN_*initial*_ is shown in Equation (17).


$$FN_{initial}=N_{GT}-TP_{initial}$$ (17)

In Equation (17), N_GT_ is the number of actual targets in the labeled dataset, TP_*initial*_ is the number of correct targets directly detected by the improved YOLOv11 model (IoU ≥ 0.5 and correct classification).

#### The testing effect analysis of cascaded network.

The open-source Common Objects in Context (COCO) dataset is used to train the YOLOv11-Pose model, and the self-built dataset are used for models testing. Taking the earplugs and gloves targets as the detection example, the testing results of the improved YOLOv11 model and the cascaded network are shown in Table 7.

**Table 7:** The testing results of the improved YOLOv11 model and cascaded network.

Detection Targets and Metrics	N_*GT*_	TP_*recovery*_	FN_*initial*_	MRR	mean
**Earlug**	12	2	6	33.3%	51.45%
**N-Earlug**	28	5	8	62.5%	
**Glove**	38	6	10	60%	
**N-Glove**	17	4	8	50%	

It can be seen from FN_*initial*_ metric in Table 7 that a significant number of missed detection for earplugs and gloves still occurred with the improved YOLOv11 model. As shown by TP_*recovery*_ metric in Table 7, some missed detection targets are successfully detected through the cascaded network, and the detection rates for both positive and negative samples of earplugs and gloves have been significantly improved.

**Fig 10:**
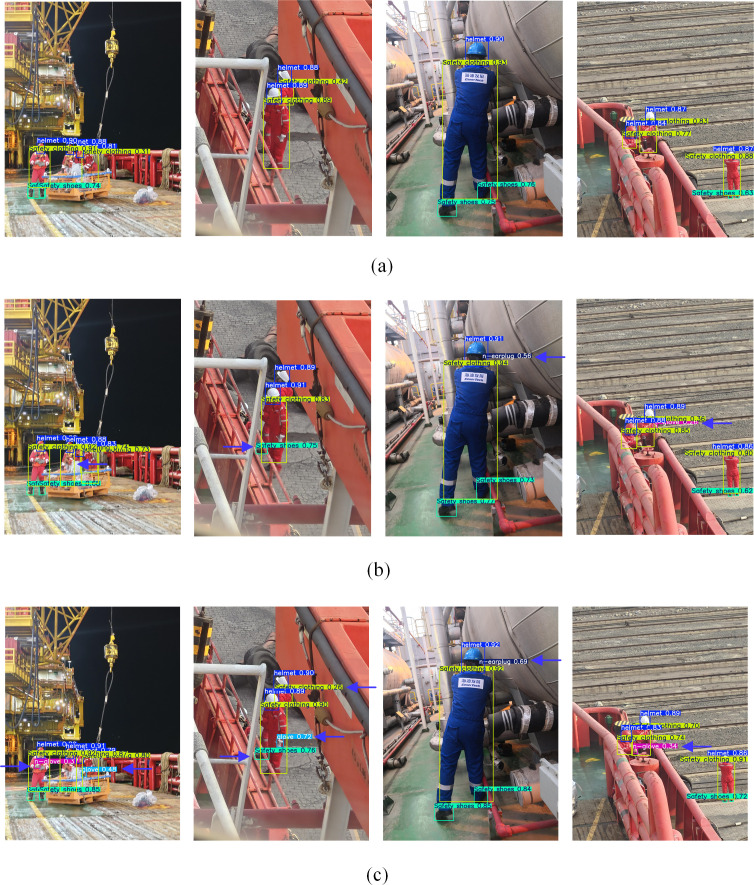
The comparison of model detection effects. (a) The original YOLOv11 model (b) The improved YOLOv11 model (c) The cascaded network.

To further verify the advantages of cascading detection, the same images are respectively input into the original YOLOv11 model, the improved YOLOv11 model, and the cascaded network for PPE targets detection. The detection results are shown in Fig 10.

As can be seen from Fig 10, comparing with the original YOLOv11 model, the improved model demonstrated higher confidence in detecting safety helmets and other targets, and the missed detection targets have been reduced. Furthermore, based on the cascaded network, the confidence of detection results for conventional targets is further improved, while the problem of missed detection for difficult detecting targets is significantly reduced, thereby verifying the superiority and effectiveness of the multi-models cascaded approach for offshore operators PPE targets detection.

## Conclusions

Based on the detecting requirement of PPE wearing condition for offshore operators, the dataset of offshore operators with PPE targets is constructed, the key issues including the optimization methods for YOLOv11 model, PPE targets detection, small-object detection, and the cascaded detection method of YOLOv11 and YOLOv11-Pose models are studied in the paper. The main researched conclusions are as follows.

1. In terms of model optimization, the SFEAF module is introduced into the backbone network of the YOLOv11 model, and the features fusion of time-domain and frequency-domain is used to enhance the recognition capability for small targets. The attention mechanism in the C2PSA module is replaced by the TSSA module to improve the model’s robustness against noise and occlusion. The original loss function is replaced by the NWD loss function to improve the model’s generalization ability and overall performance. Ablation experiments demonstrate that there are certain differences in the improvement effect of three optimization methods on the detection accuracy of different PPE targets. However, the fusion of the three optimization methods can achieve complementary advantages and overall improve the model detection accuracy for PPE targets of offshore workers.
2. For small targets detection, a secondary detection scheme based on the cascaded method of the YOLOv11 and YOLOv11-Pose models is designed. Specifically, for the small targets that can’t be detected by YOLOv11 model, the YOLOv11-Pose model is used to detect key points associated with the small targets firstly. A two points region localization method is then applied to extract the local regions of the small targets, which is subsequently re-detected using the improved YOLOv11 model. Experimental results showed that, comparing with single model detection, the cascaded network significantly reduce the missed detection rate for small targets and also improve the confidence levels of detected conventional targets.

Although the proposed cascaded scheme demonstrates strong detection performance under standard conditions, several challenges remain:

1. The robustness of the model in adverse environments, such as fog, rain, and low-light conditions, is still limited. In such cases, the degradation of visual features may lead to reduced detection accuracy, with attenuation of both image color and edge information, resulting in decreased image quality. This is especially problematic for the visibility of small personal protective equipment (PPE), such as earplugs and gloves, which are highly sensitive to environmental interference.As part of our future work, we plan to explore multispectral fusion strategies, such as combining visible light, which captures color and texture information, with thermal imaging, which provides contour and positional data of personnel. This approach aims to address these challenges and enhance the reliability of PPE detection in offshore platform environments, ultimately improving personnel safety.
2. This study primarily concentrates on the detection model architecture and the design of the NWD-based localization metric, and has not yet conducted a comprehensive quantitative evaluation of the model’s quantization and deployment on resource-constrained devices. Specifically, quantizing the model from floating-point (FP32/FP16) to INT8 introduces discretization errors, which may affect the NWD values, which are based on continuous bounding box parameters, and thus the localization performance driven by these values. Moreover, different edge hardware platforms—such as ARM CPUs, mobile GPUs, NPUs, and the Jetson series—demonstrate significant variation in their support for INT8 and runtime optimizations. Therefore, latency/throughput measurements obtained on server-level GPUs (e.g., RTX 3090) cannot be directly generalized to edge devices. To mitigate these issues, we recommend employing quantization-aware training (QAT), per-channel quantization, using a representative calibration dataset during deployment, and retaining FP32 computation for critical post-processing steps (such as NMS) to reduce cumulative errors. Given the current limitations of available hardware resources, we will address the quantization robustness of NWD and latency/memory evaluations on representative edge devices as part of our future work.

## Abbreviations

PPE: Personal Protective Equipment
DMS-DETR: Dynamic Multi-Scale Detection Transformer
LMNet: ladder-type multi-attention network
RT3DsAM: Residual Transformer 3D-spatial Attention Module
CHVG: four colored hardhats, vest, safety glass
CNN: Convolutional Neural Network
C3K2: Cross Stage Partial with kernel size 2
C2PSA: Cross Stage Partial with Spatial Attention
SPPF: Spatial Pyramid Pooling – Fast
PSA: Partial Spatial Attention
SFEAF: Spatial-Frequency Edge-Aware Fusion
FFT: Fast Fourier Transform
TSSA: Token Statistics Self-Attention
MCR2: maximum coding rate
NWD: Normalized Wasserstein Distance
P: Precision
R: Recall
AP: Average Precision
CIs: confidence intervals
MRR: Miss Recovery Rate
COCO: Common Objects in Context
